# Current Insights into Cellular Determinants of Peritoneal Fibrosis in Peritoneal Dialysis: A Narrative Review

**DOI:** 10.3390/jcm12134401

**Published:** 2023-06-30

**Authors:** Satriyo Dwi Suryantoro, Mochammad Thaha, Henry Sutanto, Sarah Firdausa

**Affiliations:** 1 Department of Internal Medicine, Faculty of Medicine, Universitas Airlangga, Surabaya 60132, Indonesia; mochthaha@fk.unair.ac.id (M.T.); henry1988md@gmail.com (H.S.); 2 Universitas Airlangga Hospital, Surabaya 60115, Indonesia; 3 Department of Internal Medicine, Faculty of Medicine, Universitas Syiah Kuala, Banda Aceh 23111, Indonesia; sarahfirdausa@usk.ac.id

**Keywords:** peritoneal fibrosis, dialysis, chronic kidney disease, simple peritoneal sclerosis, encapsulating peritoneal sclerosis, nephrology, renal replacement therapy

## Abstract

Peritoneal fibrosis is the final process of progressive changes in the peritoneal membrane due to chronic inflammation and infection. It is one of the main causes of discontinuation of peritoneal dialysis (PD), apart from peritonitis and cardiovascular complications. Over time, morphological changes occur in the peritoneal membranes of patients who use PD. Of those are mesothelial-to-mesenchymal transition (MMT), neoangiogenesis, sub-mesothelial fibrosis, and hyalinizing vasculopathy. Several key molecules are involved in the complex pathophysiology of peritoneal fibrosis, including advanced glycosylation end products (AGEs), transforming growth factor beta (TGF-β), and vascular endothelial growth factor (VEGF). This narrative review will first discuss the physiology of the peritoneum and PD. Next, the multifaceted pathophysiology of peritoneal fibrosis, including the effects of hyperglycemia and diabetes mellitus on the peritoneal membrane, and the promising biomarkers of peritoneal fibrosis will be reviewed. Finally, the current and future management of peritoneal fibrosis will be discussed, including the potential benefits of new-generation glucose-lowering medications to prevent or slow down the progression of peritoneal fibrosis.

## 1. Introduction

End-stage renal disease (ESRD) is the final stage of chronic kidney disease with a glomerular filtration rate of less than 15 mL/min [1]. In 2018, the incidence of ESRD in the United States reached 390.2 per 1 million population, while the prevalence reached 242 per 1 million individuals [2]. Renal replacement therapy (RRT) is frequently indicated to remove toxins (e.g., urea) from the blood, remove excess fluids from the body, and treat acid–base (e.g., metabolic acidosis) or electrolyte (e.g., hyperkalemia) imbalance which commonly occur in patients with ESRD. At present, two types of RRTs are often employed, particularly in situations where kidney transplantation is not an option: First, hemodialysis, in which blood is circulated through a tube and across an artificial membrane, and second, peritoneal dialysis (PD), in which dialysate can be implanted adjacent to the peritoneal membrane [3,4]. The choice to undergo hemodialysis or PD is usually based on the patient’s motivation, geographic distance from the hemodialysis unit, and patient education. Affordable treatment costs, lower risk of infection, and ease of patient mobility make PD an RRT of choice [5]. However, some complications of PD have been documented (e.g., peritonitis and malfunction of the PD system), increasing morbidity and mortality among PD users. In addition, peritoneal fibrosis can occur as a long-term complication of PD. Peritoneal fibrosis is the final process of progressive changes in the peritoneal membrane due to chronic inflammation and infection. This complication commonly occurs in patients with PD, where initial signs of fibrosis have been found in 50–80% of patients, even in the first or second year of dialysis [6]. Peritoneal fibrosis is one of the main causes of discontinuation of PD, apart from peritonitis and cardiovascular complications [7,8]. Two phases of peritoneal fibrosis can occur as a complication of PD: First, simple peritoneal sclerosis (SPS), which is characterized by peritoneal thickening, calcification, inflammation, angiogenesis, and vascular and lymphatic dilation in the absence of systemic disease, and second, encapsulating peritoneal sclerosis (EPS), a syndrome characterized by loss of ultrafiltration function, anorexia, weight loss, diarrhea, intestinal obstruction, inflammation, thickening of the peritoneum, fibrin deposition, sclerosis, calcification, and encapsulation [9,10,11,12].

The incidence of EPS varies widely, with the highest reported incidence being 19.4%, whereas more recent studies have reported lower rates ranging from 0.5% to 4.4% [13]. This high variability in event rates is due to differences in the duration of PD. For example, in the first year of PD, EPS incidence was reported as “only” 1.1%, but this figure increased to 3.4% in the third year, 8.8% in the fourth year, 9.4% in the fifth year, and 22.2% in the seventh year of PD [14,15]. The mortality rate due to this complication is also quite high. The mortality rate among PD users with EPS has been reported to range from 38 to 83%, but in more recent studies, survival may vary from 6 to 48 months or from 26 to 56%. This EPS mortality rate is also related to the duration of PD [13,15]. Because of the magnitude of the impact that peritoneal fibrosis can have on the quality of life of PD users, it is important to better understand and tackle this complication. This paper will comprehensively discuss peritoneal fibrosis in PD, from biomolecular to clinical aspects. This narrative review will first discuss the physiology of the peritoneum and PD. Next, the multifaceted pathophysiology of peritoneal fibrosis, including the effects of hyperglycemia and diabetes mellitus on the peritoneal membrane, and the promising biomarkers of peritoneal fibrosis will be reviewed. Finally, the current and future management of peritoneal fibrosis will be discussed, including the potential benefits of new-generation glucose-lowering medications to prevent or slow down the progression of peritoneal fibrosis.

## 2. The Fundamentals of Peritoneum

The peritoneum is a serous membrane that lines the peritoneal cavity. This structure has a surface area similar to that of the body, ranging from 1 to 2 m^2^ in adults, and is composed of two parts: the parietal peritoneum covering the abdominal wall and diaphragm, and the visceral peritoneum covering the intra-abdominal organs. The peritoneal cavity is lined by a single layer of mesothelial cells equipped with microvilli and covered by a thin layer of peritoneal fluid. This peritoneal fluid provides lubrication and allows free movement of the visceral organs during respiration and peristalsis [16,17]. Mesothelial cells are able to regulate inflammation and tissue remodeling through the synthesis of proinflammatory cytokines, growth factors, and matrix building blocks, as well as synthesize glycosaminoglycans and proteoglycans such as hyaluronan, decorin, syndecan-1, and perlecan which form a protective glycocalyx and provide selective permeability properties [6,16]. Mesothelial cells cover the sub-mesothelial region, which is made of a thin layer of connective tissue consisting mostly of bundles of collagen fibers with few fibroblasts, macrophages, mast cells, and blood and lymphatic vessels [12,16]. As solutes and water move across the peritoneum from the blood into the peritoneal cavity, they encounter six barriers: a layer of fluid covering the peritoneal capillary endothelium, the capillary endothelium, the endothelial basement membrane, the sub-mesothelial compact zone (SMC) which contains the interstitial space, the mesothelium, and a layer of fluid above the mesothelium [4,17].

## 3. Introduction to Peritoneal Dialysis

During PD, solutes move bidirectionally between the peritoneal capillary blood and the peritoneal cavity, mainly by diffusion and, to a lesser extent, by convection. During diffusion, solutes move down their concentration gradient from an area of high concentration to one of low concentration. The solute must be of the appropriate size and charge to pass through a semipermeable membrane. By passing fluid across the membrane in the opposite direction of blood flow, the equilibration of plasma and dialysate solute concentrations occurs. This process can remove or add solutes into the plasma fluid space depending on the relative concentrations in the dialysate and plasma. Water will also move along a gradient (in this case, an osmolar or osmotic gradient) following the solutes. Diffusion mechanisms are more effective for removing small solutes, such as serum ions and urea, than larger solutes [3,4]. For example, diffusion of urea from the capillary blood to the peritoneal cavity reaches its maximum performance at the onset of PD, when the urea concentration in the dialysate is zero. During diffusion, the concentration gradient across the peritoneal membrane slowly decreases due to the gradual movement of urea into the dialysate. In addition to the concentration gradient, other factors that affect the diffusion of solutes during PD are the total peritoneal surface area in contact with the dialysate, peritoneal vascularity, molecular weight of solutes, and intrinsic permeability of the peritoneal membrane. In clinical practice, ideally, increasing the filling volume would recruit more peritoneal membrane to contact with the dialysate, which would then increase solute clearance [4,17].

Meanwhile, convection utilizes pressure gradients (not concentration gradients, as is the case with diffusion), and their main effect is on the movement of water along with the solutes in it. The transmembrane pressure difference is increased as needed to “push” water through the membrane down the pressure gradient. This large flow of plasma fluid also “drags” the solutes in it (convective mass transfer) and forms an ultrafiltrate [3]. The amount of convective transport of this solute is determined by the transperitoneal ultrafiltration and the sieving coefficient (i.e., the fraction of solute that crosses the membrane with water flow) of the solute. Ultrafiltration in PD can be achieved either by creating an osmotic gradient across the peritoneal membrane using crystalloid agents (e.g., dextrose and amino acids) or by inducing aqueous flow with colloidal agents (e.g., icodextrin). When using crystalloid agents, the osmotic gradient is maximal at the start of dialysate filling, which decreases over time due to the dilution of the concentration of the dialysate osmotic agent and absorption of the osmotic agent into the lymphatics and tissues. This gradient can generally be maximized by using a higher concentration of dialysate fluid (e.g., a higher concentration of dextrose). In addition to the osmotic gradient, other factors that affect ultrafiltration are the hydraulic conductance of the peritoneal membrane, the effective peritoneal surface area, the reflection coefficient of osmotic agents, the hydrostatic pressure gradient, and the oncotic pressure gradient [17].

PD is commonly performed intermittently up to four times daily by injection of dialysate into the peritoneal cavity via a trans-abdominal catheter that is introduced through the anterior abdominal wall and penetrates the parietal peritoneum and with its tip placed in the pelvis. The peritoneal membrane is then used for the exchange of electrolytes, glucose, urea, albumin, and other small molecules from the blood. Ideally, dialysate fluid can be distributed throughout the peritoneal cavity and utilize the available surface area of the peritoneal membrane [18].

## 4. Peritoneal Fibrosis as a Long-Term Complication of PD

**Figure 1:**
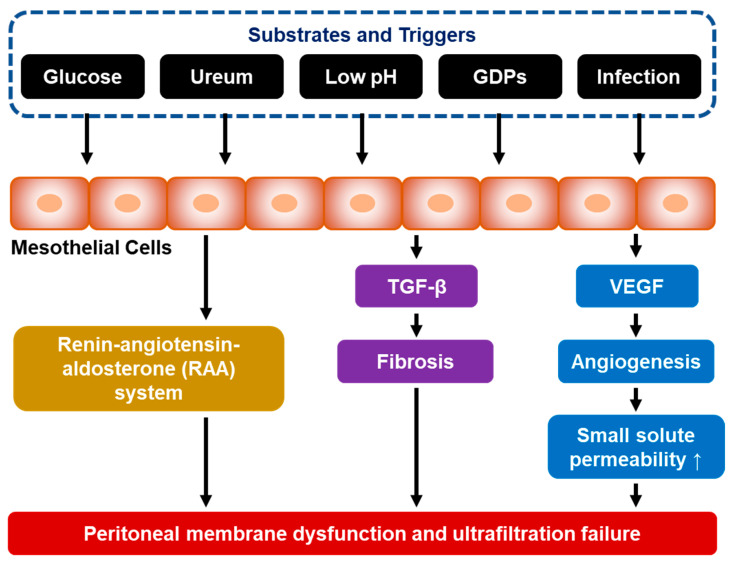
Molecular and cellular transformation of the peritoneal membrane leading to PD failure. (GDP = glucose degradation product; TGF-β = transforming growth factor beta; and VEGF = vascular endothelial growth factor).

Over time, morphological changes occur in the peritoneal membranes of patients who have used PD for a long time. Advanced glycation end products (AGEs) formed from non-enzymatic interactions between glucose residues and amino acids have been found in the mesothelial and sub-mesothelial layers in the first 3 months of PD, and play a role in the formation of irreversible cross-links between tissue proteins [6]. Long-term exposure to glucose and its glucose degradation product (GDP) induces activation of the renin–angiotensin–aldosterone system and production of a variety of proinflammatory and angiogenic factors, including nitric oxide (NO), transforming growth factor beta (TGF-β), and vascular endothelial growth factor (VEGF) (Figure 1) [4,17,19]. These factors then lead to peritoneal capillary neoangiogenesis, which will increase the effective peritoneal surface area and increase the transport of small solutes. With increased transport of small solutes across the peritoneal membrane, glucose will diffuse into the peritoneal capillaries more rapidly, resulting in a rapid loss of the osmotic gradient and a decrease in net ultrafiltration. In addition, long-term exposure to dialysis solutions that are hyperosmotic, hyperglycemic, and acidic often causes chronic inflammation and injury to the peritoneal membrane [17]. This chronic inflammation facilitates the transformation of peritoneal mesothelial cells from epithelial to mesenchymal cells (epithelial-to-mesenchymal transition [EMT] or mesothelial-to-mesenchymal transition [MMT]), which will result in mesothelial denudation, sub-mesothelial fibrosis, and impaired vascular permeability. The alteration of vascular permeability will then contribute to decreased fluid and solute transport [16,17].

## 5. The Pathophysiology of Peritoneal Fibrosis

### 5.1. Mesothelial-to-Mesenchymal Transition (MMT)

Structural changes to the peritoneal membrane, such as loss of the mesothelial cell monolayer, neoangiogenesis, sub-mesothelial fibrosis, and hyalinizing vasculopathy, are consequences of the process of repair and adaptation to inflammation. The loss of mesothelium epithelial cells caused by EMT is semi-reversible. During EMT, mesothelial cells lose cell polarization, undergo disassembly of intercellular bonds (i.e., tight junctions and adherens junctions) and, at the same time, transform into a fibroblastic form characterized by higher motility and capacity to produce and secrete extracellular matrices (ECM). With this new characteristic, mesothelial cells undergoing EMT can migrate to the sub-mesothelial zone and secrete ECM, which plays an important role in fibrogenesis [20]. Loss of cell-to-cell contact in EMT is caused by the downregulation of epithelial markers such as E-cadherin, cytokeratin, and zonula-occludens-1 (ZO-1). The downregulation of E-cadherin is due to the induction of Snail, which directly inhibits E-cadherin transcription. Proteins that make up tight junctions, such as claudin and occludin, which play a role in regulating transport in the peritoneal mesothelium, also experience expression and localization disturbances in patients with PD [20]. As a result, the integrity of the mesothelial layer barrier is disrupted, so that the mixture of dialysate fluid and the overlying peritoneal fluid (which is generally hyperosmotic, acidic, and contains GDPs and AGEs formed in the peritoneal cavity) can come into contact and enter the sub-mesothelium layer, and will cause further inflammation [4,20].

### 5.2. VEGF-Mediated Neoangiogenesis

Chronic inflammation of the peritoneal membrane will also induce neoangiogenesis or the formation of new capillaries, which increases the surface area available for the diffusion of solutes. Noteworthily, apart from EMT, VEGF also plays an important role in neoangiogenesis in the peritoneal membrane. Various proinflammatory mediators and cytokines that are increased in long-term PD use (e.g., interleukin [IL]-1b, IL-6, IL-17, TGF-β, and oxidative stress) can also increase VEGF production. For example, TGF-β is known to increase VEGF expression in mesothelial cells and fibroblasts. TGF-β inhibition was reported to reduce peritoneal fibrosis and VEGF production in animal experiments. In addition, a study demonstrated that VEGF levels decreased when patients were switched from a glucose-based PD solution to a glucose-free PD solution (i.e., icodextrin, glycerol, and amino acids), suggesting a central role of high glucose concentrations in the upregulation of peritoneal VEGF production [20].

### 5.3. The Role of TGF-β and Smad/Non-SMAD Signaling Pathways

**Figure 2:**
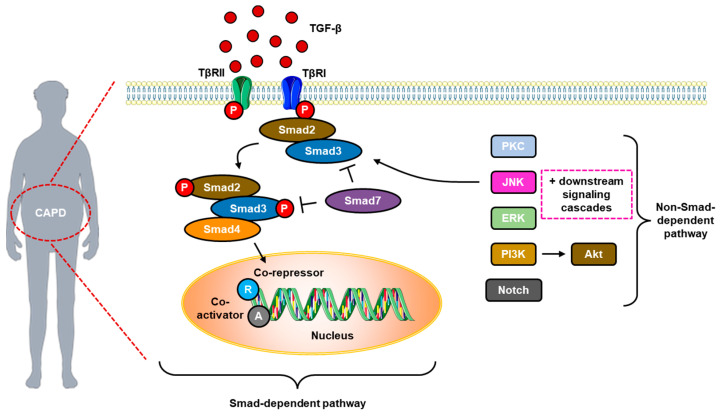
TGF-β-mediated Smad- and non-Smad-dependent pathways in peritoneal fibrosis. (CAPD = continuous ambulatory PD; ERK = extracellular signal-regulated kinase; JNK = c-Jun N-terminal kinase; PI3K = phosphatidylinositol-3-kinase; PKC = protein kinase C; TGF-β = transforming growth factor beta; TβRI = TGF-β receptor type I; and TβRII = TGF-β receptor type II.)

TGF-β has a central role in the pathogenesis of peritoneal fibrosis in PD. TGF-β facilitated by the protein kinase Cα (PKCα) signaling cascade is a common mediator of peritoneal fibrogenesis induced by glucose, GDPs, and AGEs. Exposure to dialysate with high glucose levels in mesothelial cells increases TGF-β expression through upregulation of type I and II TGF-β receptors (i.e., TGFR1 and TGFR2) in mesothelial cells [20,21]. TGF-β1 can transduce signals via Smad-dependent (classical pathway) and Smad-independent pathways, although most of the profibrotic effects of TGF-β1 travel through the Smad signaling pathway (Figure 2). In the classical pathway, Smad2/3 is phosphorylated by PKC and activated by TGFR1 and activin I-β receptor (ACTR1B). Subsequently, Smad2/3 is released from the receptor complex to form a heterotrimeric complex with Smad4 and translocate into the nucleus. Within the nucleus, these heterotrimeric complexes regulate the transcription of target genes together with various coactivators and corepressors [20]. Meanwhile, Smad7 is a type of inhibitory Smad, which inhibits Smad2/3 phosphorylation by blocking access to TGFR. Several studies have reported the benefits of Smad7 and bone morphogenic protein 7 (BMP-7) for inhibiting peritoneal fibrosis, angiogenesis, and inflammation in the long-term use of PD [20,21].

In the non-Smad signaling pathway (Figure 2), the activation of PKC, extracellular signal-regulated kinase (ERK), c-Jun N-terminal kinase (JNK), and phosphatidylinositol-3-kinase (PI3K) will activate a specific protein serine–threonine kinase, whereas high glucose levels in the PD dialysate will mediate PKC and MAPK phosphorylation. At the same time, TGF-β will modulate Akt, one of the PI3K targets, all of which will increase the risk of developing peritoneal fibrosis and EMT. In contrast, inhibition of JNK, p38 MAPK, and nuclear factor (NF)-κB was reported to counteract TGF-β-induced peritoneal fibrosis [20,21]. For example, JNK-associated leucine zipper protein (JLP) was found to have potent antifibrotic properties that counteract the profibrotic effects of TGF-β. In mice that experience peritoneal fibrosis due to dialysate induction of high glucose, there is a decrease in JLP expression, whereas JLP deletion induces the formation of peritoneal fibrosis associated with EMT, increased autophagy and apoptosis, and increased activation of TGF-β1/Smad signaling [22].

Furthermore, an increase in TGF-β will activate its downstream mediators, including connective tissue growth factor (CTGF), which plays a role in ECM production, proliferation, adhesion, and cell migration. In addition to CTGF, inflammasome NOD-like receptor protein 3 (NLRP3) and underlying proinflammatory cytokines (e.g., IL-1b) have been reported to be involved in peritoneal inflammation and fibrosis. In addition, several proinflammatory cytokines, such as IL-6 and IL-17, have also been reported to play a role in the pathogenesis of peritoneal fibrosis associated with PD [20].

### 5.4. The Role of Glucose Metabolism

Glucose metabolism also plays an important role in the pathogenesis of peritoneal fibrosis. PD patients without a history of diabetes mellitus have normal plasma glucose concentrations, but the glucose content in the peritoneal dialysate far exceeds that of severe hyperglycemia [6]. Hyperglycemia is known to increase the expression of TGF-β and hypoxia-inducible factor 1 subunit alpha (HIF-1α) by increasing the rate of glycolysis and inhibiting the pyruvate dehydrogenase complex (PDH), as well as accelerating lactic acid production. This process will increase collagen synthesis and reduce ECM degradation [20]. Hyperglycemia will cause pseudohypoxia by disrupting the cellular oxidation of nicotinamide dinucleotide (NADH) to NAD+, which will increase the NADH/NAD+ ratio. (Pseudo)hypoxia characterized by a high NADH/NAD+ ratio stimulates HIF-1 upregulation, which upregulates various factors such as erythropoietin (EPO), glucose transporter 1 (GLUT-1), VEGF, and various profibrotic and angiogenic factors, including TGF-β, plasminogen activator inhibitor-1 (PAI-1), and CTGF. Increased expression of GLUT-1 in peritoneal interstitial myofibroblasts can stimulate cellular uptake of dialysate glucose, which will increase pseudohypoxia and further stimulate GLUT-1 expression [6].

### 5.5. The Role of Epigenetics and Gut Microbiome

Epigenetic factors (i.e., deoxyribonucleic acid (DNA) methylation, histone modifications, and non-coding ribonucleic acid (RNA; i.e., microRNAs, long non-coding RNAs, and circular RNAs)) also contribute to the pathogenesis of peritoneal fibrosis [23]. For example, the miR-15a-5p microRNA is downregulated in the peritoneum of patients using PD and in human peritoneal mesothelial cells (HPMCs) stimulated with high glucose or AGEs. When a clone of this miRNA was administered to restore miR-15a-5p expression, VEGFA, which is induced by high glucose levels, could be suppressed. Consequently, inflammation and fibrosis can be suppressed in HPMCs [24,25]. Besides miR-15a-5p, peritoneal fibrosis is also modulated by miR-199a-5p, miR-214-3p, miR-30a, miR-153-3p, miR-21, miR-129-5p, miR-30b, miR-200, miR-145, miR-302c, miR-34a, and miR-29b [25,26]. Recently, the potential link between the intestinal microbiota and dysbiosis with peritoneal fibrosis has been discussed, in which several molecular and cellular mechanisms are likely to contribute to the formation of peritoneal fibrosis, for example, gut-sourced metabolites (e.g., short-chain fatty acids (SCFA) and lipopolysaccharides (LPS)), inflammation, production of reactive oxygen species and oxidative stress, activation of the host immune response, and intestinal bacterial translocation [27].

### 5.6. The Role of Macrophage Infiltration

Several studies have demonstrated the importance of macrophage infiltration in the pathogenesis of peritoneal fibrosis. An experimental study showed that monocyte and macrophage depletion provided less structural changes, less MMT, less fibrin deposition, and less interstitial fibrosis. Moreover, in macrophage-depleted animals, there were reductions in α-SMA and fibronectin, as well as an elevation of e-cadherin. Overall, this study concluded that peritoneal fibrosis was inhibited in the absence of macrophages, whereas the addition of M1 macrophage resulted in structural damage, ECM deposition, and ultrafiltration failure. Such detrimental effects of M1 macrophage were further associated with an increased expression of TLR4 [28]. Another study also demonstrated the role of M2 macrophage in peritoneal fibrosis. A complete depletion of M2 macrophage using liposome-encapsulated clodronate, a specific macrophage scavenger, reduced the expression of CD206 and TGF-β, and improved peritoneal fibrosis by reducing the thickness of the peritoneal membrane, protecting mesothelial cells from transformation and reducing the expression of type I collagen and fibronectin [29]. 

### 5.7. Peritoneal Remodelling in Diabetes Mellitus

A previous study concluded that patients with diabetes mellitus with high levels of urea (i.e., uremic) tend to have a thicker peritoneum (148.9.0 ± 106.8 μm) than patients without diabetes (107.0 ± 69.6 μm), although this difference did not reach statistical significance. Hyalinizing vasculopathy is also more common in uremic patients with diabetes than in uremic patients without diabetes, accompanied by thickening of the blood vessel walls in the postcapillary venules. As a result, there is a decrease in the ratio of luminal-to-vessel diameter (L/V) in uremic patients with diabetes compared to uremic patients without diabetes. In general, diabetes can increase the formation of AGEs in the peritoneal blood vessels, which will induce vasculopathy in the peritoneum [30].

Meanwhile, experimental studies on rats injected with streptozotocin (STZ) to induce diabetes mellitus reported that peritoneal damage had occurred 21 days after STZ injection, where diabetes increased the thickness of the sub-mesothelium layer. Mesothelial cells in diabetes also have shorter microvilli without a decrease in microvilli density compared to the control group. Furthermore, in diabetes, there is a decrease in the expression of pan-cadherin and cytokeratin which are markers of epithelial cells, as well as an increase in α-SMA, which is a marker of mesenchymal cells, indicating an increase in MMT in diabetes. In addition, diabetes also modulates the expression of tight junction proteins (i.e., claudin and occludin) in the peritoneum and increases oxidative stress in the peritoneal cavity. This tight junction modulation causes the peritoneum in diabetes to be more permeable to solutes. Overall, the findings of this study indicate that the efficiency of PD in diabetics depends on the preexisting remodeling of the peritoneum prior to PD, and diabetes can cause damage to the peritoneal membrane, even in non-uremic conditions [31].

### 5.8. Expressions of Glucose Receptors SGLT-2, GLUT, and DPP-4 in Peritoneum and Their Roles in the Formation of Peritoneal Fibrosis

Generally, glucose transport in the body occurs via two types of glucose transporters: via solute carrier family 2 (SLC2A) or glucose transporter (GLUT), and via solute carrier family 5 (SLC5A) or sodium–glucose cotransporter (SGLT). GLUT is predominantly expressed in enterocytes in the intestine and kidney, as well as in many other organs. In contrast, SGLT expression is restricted to the small intestine, renal proximal tubule, and mesangial cells. In human peritoneal biopsies, expression of SGLT-2, GLUT-1, and GLUT-3 was found, where SGLT-2 protein expression increased with the duration of PD and in patients with EPS. This finding could be related to the effect of high glucose levels in dialysis fluids and diabetes on the pathological changes in the peritoneal membrane that occur in peritoneal fibrosis, including EPS [32]. Recently, a study reported changes in the expression of glucose transporters in the peritoneum due to diabetes mellitus. In diabetic rats, there was an increase in SGLT-1, SGLT-2, and GLUT-2 expression, as well as a decrease in GLUT-1 expression when compared to the control group. This suggests that the expression of glucose transporters in peritoneal mesothelial cells is influenced by glucose concentration, which will then affect the response to intraperitoneal inflammation, regulation of matrix protein production, or peritoneal fibrosis during PD [31].

In addition to the glucose transporter, the dipeptidyl peptidase IV receptor (DPP-4) also contributes to the pathophysiology of PD-associated peritoneal fibrosis. DPP-4 is a type II integral membrane glycoprotein with serine peptidase activity that degrades incretins, for example, glucagon-like peptide-1 (GLP-1). DPP-4 is widely expressed on the surface of various cell types, including endothelial cells, renal epithelial cells, immune cells, and mesothelial cells [33]. At increased glucose concentrations, for example, in diabetes, DPP-4 protein expression increases with increasing MMT. Meanwhile, GLP-1 expression decreased inversely proportionally to the increase in DPP-4 in diabetes mellitus. Glucose exposure also activates phosphorylated Smad3, which upregulates Snail, followed by increasing mesenchymal markers (i.e., collagen type I, fibronectin, α-SMA, vimentin, and Twist) and decreasing epithelial markers (i.e., ZO-1). In addition, exposure to glucose also increases the activation of the NF-κB signaling pathway and increases oxidative stress, and reduces levels of natural antioxidants in the body [33].

### 5.9. TGF-β and VEGF in Hyperglycemia

**Figure 3:**
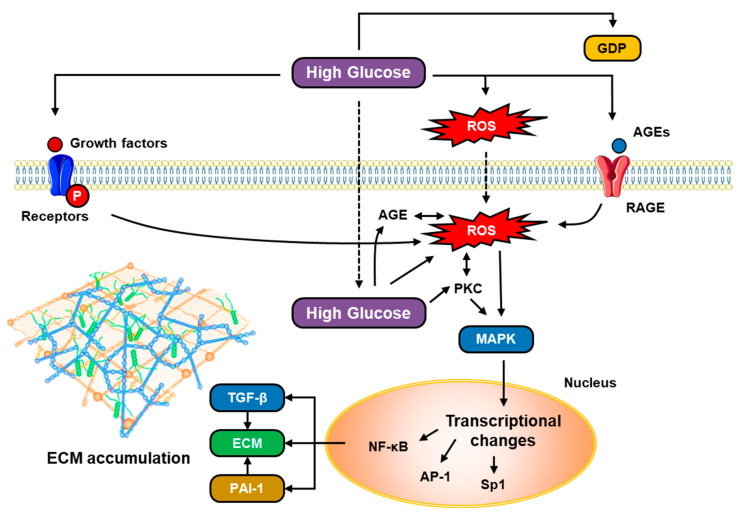
High glucose concentration (hyperglycemia) promotes extracellular matrix formation in peritoneum mesothelial cells (AP-1 = activator protein 1; AGEs = advanced glycation end products; ECM = extracellular matrix; GDP = glucose degradation product; MAPK = mitogen-activated protein kinase; NF-κB = nuclear factor kappa B; PAI-1 = plasminogen activator inhibitor-1; PKC = protein kinase C; RAGE = advanced glycation end product receptor; ROS = reactive oxygen species; and TGF-β = transforming growth factor beta).

High glucose levels (hyperglycemia) will stimulate fibronectin and TGF-β mRNA and protein expression in peritoneal mesothelial cells, as well as increase procollagen peptides secreted by mesothelium cells, inducing peritoneal fibrosis [34]. Activation of TGF-β and VEGF by hyperglycemia is closely related to activation of the diacylglycerol (DAG)–PKC signaling pathway (Figure 3). In conditions of hyperglycemia and diabetes, there is an increase in the glycolytic intermediary dihydroxyacetone phosphate, which will be reduced to glycerol-3-phosphate, and then an increase in DAG synthesis de novo. DAG is one of the PKC activating substrates besides cellular oxidants and AGEs, while PKC is a group of enzymes that regulate various cell functions and are associated with many intracellular signaling pathways, including the expression of proinflammatory cytokines such as VEGF, platelet-derived growth factor (PDGF), endothelin-1, vascular cell adhesion molecule (VCAM) and intercellular adhesion molecule (ICAM), as well as TGF-β [34,35]. PKC activation will induce transcription of c-fos and c-jun, which are proto-oncogenes that regulate gene transcription through the AP-1 activating protein. Because the promoter region of TGF-β and fibronectin has AP-1, PKC can be an upstream signaling molecule of TGF-β [34]. As a result, hyperglycemia can increase TGF-β and facilitate the activation of Smad/non-Smad signaling pathways in the formation of peritoneal fibrosis.

Meanwhile, high glucose levels will produce AGEs which have important roles both outside and inside cells. It was stated that intracellular AGE formation increased significantly in endothelial cells after 1 week in a hyperglycemic environment. Circulating AGEs can interact with AGE receptors (RAGEs) in the endothelium, causing changes in cell function, for example, upregulation of the transcription factor NF-κB. Activation of RAGEs by AGEs will also transduce many signals (e.g., NAD[P]H oxidase, p21ras, mitogen-activated protein kinase [MAPK], extracellular signal-regulated kinase 1/2 [ERK1/2], mitogen p38, and GTPase Cdc42 and Rac) which activate and translocate nuclear transcription factors, including NF-κB, which transcribe their target genes, such as endothelin-1, VCAM-1, ICAM-1, E-selectin, tissue factor, thrombomodulin, VEGF, and proinflammatory cytokines, including IL-1α, IL-6, and TNF-α, and RAGE itself.

## 6. Diagnostic Markers of Peritoneal Fibrosis in PD

An ideal peritoneal fibrosis biomarker should be directly accessible in dialysate effluent to enable the identification of patients with PD who are at high risk of complications [20]. One of the inflammatory markers in dialysis fluid that is currently used is IL-6 levels in dialysis effluent. IL-6 levels are increased in the effluent of patients with subclinical bacterial infections and acute bacterial peritonitis and can be used to evaluate bacterial clearance during infection [20,36]. Besides IL-6, IL-1b and IL-17 also have the potential to be markers of inflammation in the peritoneum. The European Training and Research in PD Network (EuTRiPD) consensus addresses several available and accessible biomarkers from dialysis effluent, such as IL-6, IL-8, CA-125 (which are markers of EMT), and advanced oxidized protein products (AOPPs), as well as potential new biomarkers such as inflammatory markers (e.g., IL-17, M1/M2 macrophages, regulatory T cells [Treg], and Th17 helper T cells), MMT markers (e.g., miRNA-21 and miRNA-31) and markers of cell senescence/response to cellular stress [36]. Th17-mediated inflammatory responses, particularly the cytokine IL-17, have recently been shown to play a central role in peritoneal damage. Experimental modulation of the Th17 response and/or enhancement of the Treg response may preserve peritoneal membrane function. Thus, chronic inflammatory damage to the peritoneal membranes may be modulated, at least in part, by regulating Th17/Treg balance [36]. Peritoneal mesothelial cells exposed to PD fluid for a long time show a phenotype similar to cell senescence, which is generally characterized by irreversible growth arrest, distorted cell morphology, and altered cytokine secretion (including increased release of IL-6 and chemokine motif CXC-8 (CXCL-8)). In addition, high glucose levels in the dialysate fluid cause oxidative stress, which accelerates cell aging. Thus, the presence of cell aging markers, e.g., AOPPs, in the dialysate fluid may indicate aging and damage to the peritoneal mesothelial cells. Furthermore, levels of cellular stress response markers, such as heat shock proteins (HSPs) Hsp27 and Hsp72, are also affected by dialysate fluid toxicity and inflammation. In mesothelial cell cultures derived from effluent from PD patients, the expression of Hsp27 and Hsp72 is affected by the MMT process [36]. Several prospective cohort studies reported that levels of matrix metalloproteinase 2 (MMP-2), PAI-1, and VEGF in dialysate fluid positively correlated with the duration of PD usage and can also be used as a marker of peritoneal fibrosis in PD patients [6,37,38,39].

Several other molecular markers are also mentioned as having the potential to be used in the diagnosis and monitoring of peritoneal membrane inflammation, for example, microRNA and aquaporin-1 (AQP1), which are excreted by mesothelium cells [6,20]. Secreted protein acidic and rich in cysteine or SPARC (osteonectin), monocyte chemoattractant protein (MCP)-1, and soluble cluster differentiation (sCD)-59 were also identified as potential biomarkers in PD effluent, where their increase is associated with peritoneal membrane failure and death [40,41]. Another study documented the potential of effluent decoy receptor 2 (eDcR2) as a biomarker of peritoneal fibrosis in PD patients. DcR2 is mainly expressed in peritoneal fibroblasts and co-localized with α-smooth muscle antigen (α-SMA), vimentin, collagen I, and fibronectin [42]. In addition to eDcR2, serum α-Klotho below 742 pg/mL is also associated with the sub-mesothelial thickness of the peritoneal membrane, whereas galectin-3 uremic levels are associated with PD failure and time to PD failure [43].

## 7. Managements of Peritoneal Fibrosis in PD

As explicated above, the role of PD dialysate in the pathophysiology of peritoneal fibrosis is vital. More biocompatible PD solutions are preferred to slow down the progression of peritoneal fibrosis. Those solutions include fluids with neutral pH, low GDPs, low lactate, bicarbonate buffer, normal osmolarity (e.g., icodextrin), and other improved solutions that do not induce (low-grade) inflammation or injure the peritoneal membrane. A Cochrane systematic review of 42 studies showed that biocompatible PD fluids could improve peritoneal function (i.e., D/P creatinine ratio), residual renal function, and residual urine volume [44]. In addition to selecting appropriate and more biocompatible dialysate fluids [6,20], several molecular targets can also be modified with the aim of inhibiting the process of peritoneal fibrosis formation in PD. For example, several studies have reported the potential benefit of inhibiting Toll-like receptors (TLRs), particularly TLR2 and TLR4, to mitigate the formation of peritoneal fibrosis associated with PD through modulation of macrophage function and mesothelial cell response to dialysate fluid and infection [45]. Soluble TLR2 (sTLR2), a TLR inhibitor, can inhibit inflammation of the peritoneal membrane by acting both as a decoy molecule that will bind to TLR2 ligands, as well as a molecule capable of modulating the activity of the CD14 coreceptor, which is the main coreceptor of TLRs. In addition, the use of anti-TLR2 and anti-TLR4 antibodies has also been shown to suppress inflammation of the peritoneal membrane in preclinical studies [45].

A number of pharmacological therapies target the inflammatory components that precipitate PD, including corticosteroids, colchicine, azathioprine, cyclosporine, mycophenolate mofetil (MMF), and inhibitors of the mammalian target of rapamycin (mTOR). Of these, corticosteroids are the most studied, and although mTOR inhibitors and MMF are said to have a theoretical advantage in improving fibrosis, this is speculative and largely confined to post-kidney transplant patients with additional indications for immunosuppressant administration [11]. Meanwhile, tamoxifen is a selective estrogen receptor modulator (SERM) with strong antifibrotic properties, which is achieved through the inhibition of TGF-β, a key cytokine in the pathogenesis of peritoneal fibrosis [11]. In addition to tamoxifen, new pharmacological agents such as nintedanib are also reported to prevent TGF-β1-related fibrosis. Nintedanib can prevent TGF-β1-induced E-cadherin downregulation, reduce angiogenesis, and suppress cytokine production in the peritoneum [46]. An experimental study reported that administration of nintedanib reduced chlorhexidine gluconate-induced peritoneal fibrosis and reduced expression of collagen I and fibronectin. At the same time, there is phosphorylation of the platelet-derived growth factor receptor (PDGF), fibroblast growth factor receptor (FGFR), VEFG receptor (VEGFR), and the Src family of kinases. Nintedanib inhibits EMT by decreasing the expression of α-SMA and vimentin, as well as preventing the decreased expression of E-cadherin in peritoneal membranes exposed to chlorhexidine gluconate and in HPMCs (in vitro) exposed to TGF-β1. Nintedanib also suppresses the expression of Snail and Twist, two transcription factors associated with EMT. In addition, nintedanib therapy inhibited the expression of several cytokines/chemokines, including tumor necrosis factor alpha (TNF-α), IL-1b and IL-6, MCP-1, and the prevented macrophage infiltration of injured peritoneal membranes. In addition, nintedanib can also reduce peritoneal neovascularization induced by chlorhexidine gluconate [47]. Next, a recent study also demonstrated the potential of VEGF inhibition in preventing peritoneal fibrosis. Tetramethylpyrazine, a VEGF/Hippo/YAP inhibitor, was shown to reduce neoangiogenesis in PD and prevented peritoneal fibrosis in mice and in human peritoneal vascular endothelium, one of which was by attenuating the VEGFR translocation from the trans-Golgi network to the cell membrane [48]. 

In addition to pharmacological agents, adipose-derived mesenchymal stem cells (ADSCs) can also slow the development of peritoneal fibrosis induced by PD through the polarization of M2 macrophages [49]. Similarly, serum free-human mesenchymal stem cells (SF-MSCs) inhibited ECM deposition, inflammatory cell deposition, and mesenchymal cell transformation [50]. Meanwhile, genetic and epigenetic modification have also emerged as potential alternative therapeutic modalities for peritoneal fibrosis [51,52,53]. For example, enhancer of zeste homolog 2 (EZH2), a histone-lysine N-methyltransferase enzyme that catalyzes the addition of a methyl group to histone H3 of lysine 27 [54], was detected in large amounts in the peritoneum of patients with associated peritonitis PD and dialysis waste in long-term PD patients. The amount of EZH2 positively correlated with the expression of TGF-β1, VEGF, and IL-6. Inhibition of EZH2 with 3-deazaneplanocin A (3-DZNeP) reduces peritoneal fibrosis and inhibits the activation of several profibrotic signaling pathways, including TGF-β1/Smad3, Notch1, EDGF receptors, and Src. EZH2 inhibition also inhibits STAT3 and NF-κB phosphorylation, and reduces lymphocyte and macrophage infiltration and angiogenesis in injured peritoneum. In HPMCs, 3-DZNeP was able to suppress TGF-β1-induced upregulation of α-SMA and collagen type I and maintain E-cadherin levels [53]. Meanwhile, inhibition of EZH2 with GSK343 can reduce lipid deposition on the peritoneal membrane induced by high glucose levels, and also prevent EMT and peritoneal fibrosis through modulation of klotho expression [55].

## 8. Peritoneal Rest in PD-Associated Peritoneal Fibrosis

Peritoneal rest has been recommended to prevent, slow down or even reverse peritoneal fibrosis [46,56]. Peritoneal rest is an attempt to stop the continuous exposure of the peritoneum to dialysate fluid which can cause MMT in peritoneal mesothelial cells. In several intervention studies, peritoneal rest can restore ultrafiltration capacity, reduce permeability, and restore morphological changes in the peritoneal membrane, such as parietal peritoneal thickening, mesenteric fibrosis, and angiogenesis [57,58,59]. Peritoneal rest has been shown to improve fibrosis, and during peritoneal rest, patients are temporarily switched to hemodialysis [46]. To increase the effectiveness of this therapy, peritoneal rest must be performed immediately after changes in peritoneal membrane permeability occur [59].

## 9. Potential Roles of New-Generation Glucose-Lowering Drugs to Limit PD-Associated Peritoneal Fibrosis

Incretin-based diabetes mellitus therapies such as DPP-4 inhibitors (e.g., sitagliptin) and GLP-1 receptor agonists (e.g., exendin-4) are new-generation diabetes mellitus pharmacotherapies that are frequently used in clinical practice. An experimental study reported that sitagliptin and exendin-4 significantly improved MMT and suppressed glucose-induced actin stress fiber formation. Sitagliptin and exendin-4 also inhibited DPP-4 protein expression, the activity of soluble DPP-4, phosphorylated Smad3, and activated Snail due to glucose exposure. Expression of glucose-upregulated mesenchymal marker proteins such as type I collagen, fibronectin, vimentin, and α-SMA was also significantly decreased by sitagliptin and exendin-4 therapy. Both also inhibit the TGF-β/Smad3 pathway and its downstream signaling cascades, and suppress the expression of NF-κB and NOX-1, all of which protect mesothelium cells against cell damage due to hyperglycemia and diabetes mellitus [33].

Other studies demonstrated the potential for SGLT-2 inhibition in peritoneal fibrosis. Dapagliflozin, an SGLT-2 inhibitor, significantly inhibits the uptake of glucose from dialysate via the SGLT-2 transporter in the peritoneal membrane, reducing TGF-β concentrations in dialysis effluent, peritoneal thickening and fibrosis, and microvessel density, which in turn increases ultrafiltration [60,61]. A randomized clinical trial reported that SGLT-2 inhibitors increased TGF-β excretion in the urine in patients with type 2 diabetes mellitus with albuminuria [62]. Interestingly, another study stated that SGLT-2 inhibitors did not affect glucose transport across the peritoneal membrane [63]. In contrast, follow-up studies suggest that phloretin, a non-selective GLUT inhibitor, can inhibit glucose transport and increase ultrafiltration efficiency in PD [64,65].

## 10. Peritoneal Function Test as a Non-Invasive Modality to Monitor Peritoneal Membrane Quality

Peritoneal function tests (PFT), for example, the peritoneal equilibration test (PET), are non-invasive approaches to semi-quantitatively assess the performance of PD. The PET measures the rate of transfer of solutes and water across the peritoneal membrane. It is performed by analyzing the contents of blood and dialysis effluent at specific times during the PD dwell. There are at least three parameters measured in a PET: dialysate to plasma (D/P) ratio of solutes (e.g., creatinine, electrolytes, urea, phosphate, and protein), time-specific dialysate glucose to initial dialysate glucose ratio (Dt/D0 glucose), and ultrafiltration volume (UFV) [66,67]. Sample collections are usually conducted at several time points (e.g., every hour) up to 4 h of dwell time. Subsequently, the D/P and/or Dt/D0 are calculated and plotted over the population reference values. For example, in 1990, Twardowski classified patients’ peritoneal transport into four groups: low, low average, high average, and high, with a mean value of D/P creatinine ratio of 0.65 and a mean UFV of 2368 [68]. A high or high average PET score indicates a rapid transport equilibrium, whereas a low or low average score indicates a slow transport equilibrium. A previous study showed that biocompatible PD solution (i.e., a neutral solution with low GDPs), as compared to non-biocompatible PD fluid (i.e., an acidic solution containing high GDPs), yielded less AGE accumulation, vascular sclerosis, and peritoneal fibrosis, with a lower PET score, indicating more effective ultrafiltration [69]. On the contrary, the high transporter group had an increased sub-mesothelial fibrous layer, a hallmark of MMT [70]. 

## 11. Summary

Peritoneal fibrosis is a late-stage complication that occurs with prolonged use of PD. Pathophysiologically, EMT/MMT, neoangiogenesis, and sub-mesothelial fibrosis are three important processes in the remodeling of the peritoneal membrane due to chronic inflammation. TGF-β, VEGF, Smad, and non-Smad signaling pathways have been reported to be involved in the pathogenesis of peritoneal fibrosis, and are upregulated in conditions of hyperglycemia and diabetes mellitus. Until now, the search for accurate peritoneal fibrosis biomarkers is ongoing, e.g., IL-6, IL-8, CA-125, AOPPs, IL-17, M1/M2 macrophages, Treg/Th17, microRNA, and Hsp. Meanwhile, the prevention and treatment of peritoneal fibrosis include the selection of biocompatible dialysate fluid, prevention of infection and chronic inflammation, and the use of pharmacological agents that can inhibit the signaling molecules involved, for example, TGF-β inhibition by tamoxifen and nintedanib. Several new-generation antidiabetic drugs (e.g., SGLT-2, GLUT, and DPP-4 inhibitors) are deemed to be able to improve peritoneal fibrosis, although their clinical application as a treatment modality for peritoneal fibrosis still requires further research.

## Data Availability

Not applicable.
